# Indian Clinician’s Perspective on the Approach to the Management of Hypertension: A Cross-Sectional Study

**DOI:** 10.7759/cureus.57435

**Published:** 2024-04-01

**Authors:** C Venkat S Ram, A Muruganathan, Manjula S, Krishna Kumar M

**Affiliations:** 1 Hypertension and Blood-Pressure Management Clinic, Apollo Hospitals, Hyderabad, IND; 2 Department of Public Health, Shristy AG Hospital, Tirupur, IND; 3 Department of Medical Services, Micro Labs Ltd, Bengaluru, IND

**Keywords:** sedentary lifestyle, antihypertensive, hypertension, management, perspective

## Abstract

Introduction

Hypertension (HTN) is considered one of the most frequent life-threatening noncommunicable illnesses. Because HTN has a significant public health impact on cardiovascular health status and healthcare systems in India, it is critical to study Indian clinicians' approaches to HTN management.

Methodology

This was a cross-sectional, multicentric, non-interventional, and single-visit study that aimed to gather data from across India and examine sociodemographic characteristics and clinician treatment choices in the management of HTN in Indian individuals. As a result, building an information platform about HTN is critical to preventing and controlling this growing burden.

Results

A total of 5298 patients were recruited in the study from 1061 study centers across India. Among the study patients, 66.67% were females with a mean age of 53.95 ± 14.4, and 66.28% of hypertensive patients presented comorbidities. Among the known risk factors for HTN, 2227 (44.5%) were smokers, while 2587 (51.7%) had sedentary lifestyles. A family history of HTN in either one or both parents was seen in 1076 (21.50%) patients. In management, 40.40% of patients were on anti-hypertensive monotherapy. Amlodipine (41.8%) in monotherapy and amlodipine + metoprolol (32.34%) in combination therapy were the most commonly prescribed antihypertensive.

Conclusion

Management of HTN can be improved by imparting patient education and awareness about the need for medication compliance, lifestyle modifications, and regular follow-up clinic visits.

## Introduction

Hypertension (HTN) is a major risk factor for cardiovascular diseases globally and a leading cause of premature death and disability [1]. It is a developing concern in India, putting a tremendous strain on the healthcare system. The HTN epidemic in India is worsened further by the fact that a huge part of the population is clueless about their HTN status, hence known as a “silent killer” [2]. Standardizing HTN management through an evidence-based model that sets thresholds for diagnosis, treatment goals, follow-up intervals, and choice of drugs can lead to improved management of HTN [3]. Achieving a better HTN control rate at the population level is critical in reducing cardiovascular morbidity and mortality [4]. Medication adherence in hypertensive patients is important because HTN is a disease that is not curable; therefore, it must always be controlled to avoid complications that can lead to death [5].

Despite the significant prevalence of HTN in India, knowledge, treatment, and management are lacking in both urban and rural populations. This could be the result of factors like low educational status, poverty, and rural residence, as well as physiological factors like obesity [6]. Several medications are effective as well as safe for the treatment of HTN. However, due to the availability of multiple drugs and varying levels of awareness about the national and international guidelines amongst practitioners in India, the achievement of the BP target becomes challenging [7]. When it comes to the need to have correct and reliable information on various parameters, including morbidity and mortality, a robust registry system seems to be the only logical solution [8].

The study was undertaken to assess the HTN trends in India to pool data from across India and evaluate the sociodemographic variables and clinician management preferences in the treatment of HTN in the Indian population. Thus, this created an information platform about HTN, which is critical for preventing and controlling this rising burden on the healthcare system.

## Materials and methods

This is a cross-sectional, multicentric, non-interventional, and single-visit study. The sample size of 3600 was calculated considering the prevalence for HTN in India as 29.8% with 5% relative precision, 95% confidence interval, and 80% power. A total of 1061 sites across India participated in the study during 2021-2022.

Patients aged 18-80 years old with known or freshly diagnosed HTN under treatment and with regular follow-ups with the clinicians were included in the study following the informed consent signed by the willing patients. The blood pressure was measured in a sitting position for each three times consecutively and the average of three measurements was recorded. Patients who required hospitalization for any reason, as well as those who were pregnant or lactating, were excluded from the study.

After receiving approval from the Bangalore Ethics Committee with reference ECR/355/Indt/KA/2022, the patients were recruited during the regular follow-ups to the clinicians. The investigator collected the socio-demographic data and history along with blood pressure readings of the patients on the data collection form. The data underwent descriptive statistical analysis for demographic characteristics and the study results are summarized with mean and standard deviation (SD) for continuous variables along with frequency and percentages wherever required. The study endpoints included demographic variables, medical history, vital parameters, treatment history, current treatment, concomitant medications used, and adherence to the treatment. The analysis population consisted of all enrolled individuals.

## Results

A total of 5298 patients were recruited in the study from 1061 study centers across India. Among the 5298 case record forms, 5004 forms were filled and were collected by study coordinators. Forms that were left blank or incomplete were not included in the analysis.

**Figure 1:**
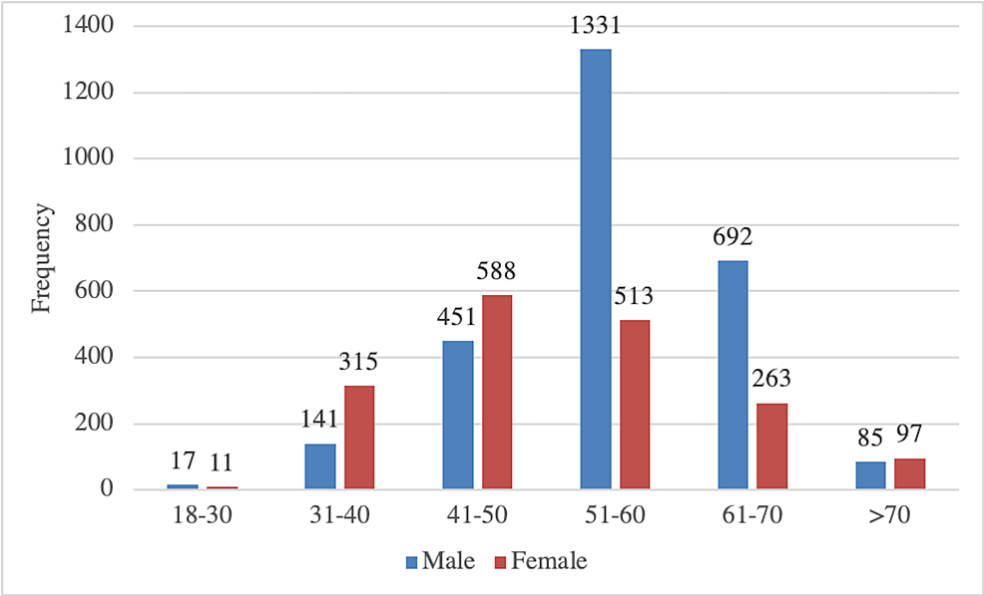
Age and gender-wise distribution of recruited patients

Among the study patients, 66.67% were females while 33.32% were males. The mean age of the recruited patients was 53.95 ± 14.4. The patient’s age group distribution is shown in Figure 1. The mean height of the patients was 163 ± 13 cm. Of the patients, 537 (10.62%) were obese while 4467 (89.38%) patients were within normal weight with a mean weight of 66.3 ± 8.7, and mean BMI was 25.5 ± 5.19 kg/m2. Of the study patients, 80.9% were from urban areas, while 19.1% were from rural areas.

The analysis of personal history stated that the study population consisted of 3797 (75.88%) participants following the non-vegetarian diet type and 1207 (24.12%) following the vegetarian diet type. The factors like the use of computers and smartphones are included in Table 1.

**Table 1:** Distribution of participants based on personal history and lifestyle factors

Factors			Personal history	N (%)
Type of diet			Vegetarian diet	1207 (24.12%)
			Non-vegetarian diet	3797 (75.88%)
Screen time				
Type		N (%)	Screen time/day (mean ± SD)	
Computer use	Yes	1646 (32.90%)	3 hours ± 15 minutes	
	No	3358 (67.1%)		
Smartphone use	Yes	3353 (67%)	4 hours ±17 minutes	
	No	1651 (33%)		

**Figure 2:**
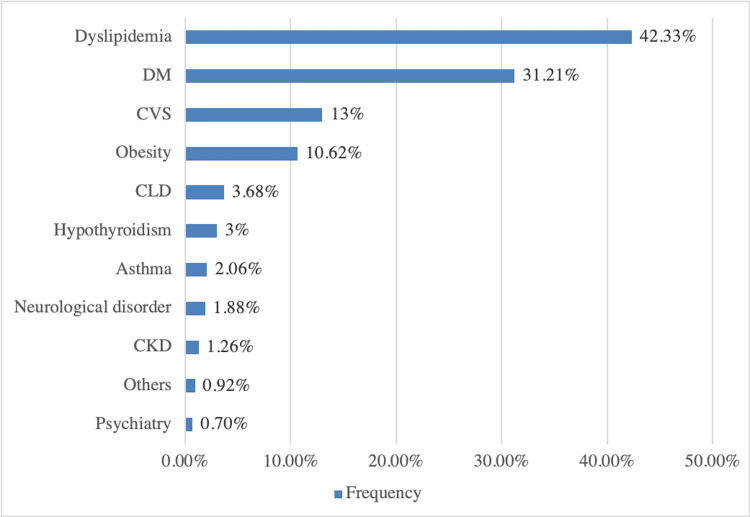
Comorbidities in hypertensive patients CKD = chronic kidney disease; CLD = chronic liver disease; CVS = cardiovascular; DM = diabetes mellitus.

Comorbidities were present in 66.28% of hypertensive patients. Amongst various comorbidities, dyslipidemia was most common (42.33%), followed by diabetes mellitus (31.21%) and cardiovascular disease (13%), as shown in Figure 2.

Table 2 demonstrates the associated risk factors with hypertension stating that 2227 (44.5%) were smokers and the mean duration of smoking was 10.5 ± 1.3 years. Regular exercisers were undertaken by 2417 (48.30%), with an average duration of exercise of 43 ± 8 minutes every day. Habitual alcohol drinkers were 1892 (37.80%), with a mean duration of alcohol drinking of 11.15 ± 7.2. A family history of HTN in either one or both parents was seen in 1076 (21.50%) patients.

**Table 2:** Risk factors associated with hypertension

Habits	Remark	N (%)
Smoking habits	Yes	2227 (44.5%)
	No	2777 (55.5%)
Alcohol use	Yes	1892 (37.80%)
	No	3112 (62.2%)
Exercise	Yes	2417 (48.30%)
	No	2587 (51.7%)
Family history	Yes	1076 (21.50%)
	No	3928 (78.5%)

**Figure 3:**
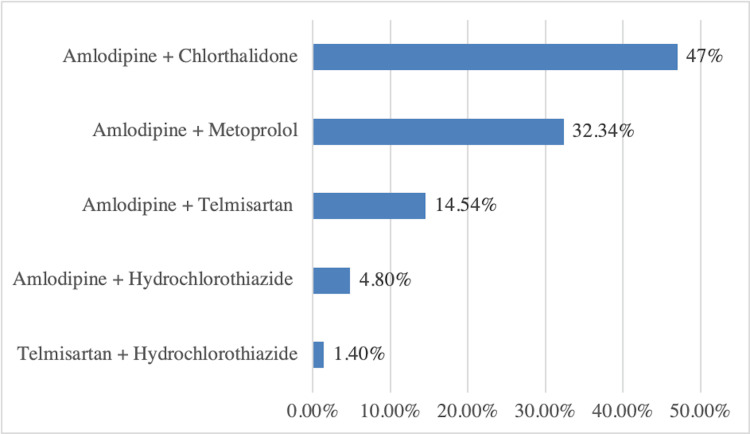
Combination antihypertensive therapy

Of the patients, 40.40% were on antihypertensive monotherapy while the rest were on combination therapy. Amongst monotherapy, amlodipine (41.8%) was most prescribed, followed by telmisartan (32.6%), cilnidipine (23.9%), enalapril (1%), and metoprolol (0.77%). Amlodipine + chlorthalidone (47%) was the most prescribed antihypertensive combination followed by amlodipine + metoprolol (32.34%), as shown in Figure 3. The distribution of patients based on diagnosis, compliance, and treatment goals is illustrated in Table 3.

**Table 3:** Distribution of patients based on the diagnosis, compliance, and treatment goals

Clinical disposition factors		Total No. of patients, N (%)	Men, N (%)	Women, N (%)
Diagnosis	New diagnosis	1632 (32.6%)	1074 (65.8%)	558 (34.2%)
	Known cases of hypertension	3372 (67.4%)	2014 (59.73%)	1358 (40.27%)
Compliance	Yes	2117 (42.3%)	1238 (58.47%)	879 (41.53%)
	No	2887 (57.7%)	1602 (55.5%)	1285 (44.5%)
Treatment goal	Diabetic (130/80 mm Hg)	1562 (31.21%)	1013 (64.8%)	549 (35.2%)
	Nondiabetic (140/90 mm Hg)	3437 (68.79%)	1828 (53.2%)	1609 (46.8%)

The concomitant drugs like metformin (1541, 98.6%) in antidiabetics, rosuvastatin (1282, 60.56%) in antilipidemic, and Ecosprin (304, 99.8%) in antiplatelets were the most commonly prescribed.

## Discussion

The study reported a preponderance of male gender and adult age group among study participants, which is in line with the study conducted by Rachana et al. (2014) [3]. However, a study conducted by Katibeh et al. (2020) [9] reported female preponderance. HTN is now regularly found in young individuals between the ages of 18 and 40 years. This could be credited to a variety of risk factors like the habit of smoking, alcohol use, sedentary lifestyle, and obesity. As per the study conducted by Kordvarkane et al. (2023) [10], smoking affects the flow properties of blood and arterial wall behavior leading to arterial stiffness. In the study conducted by Briasoulis et al. (2012) [11], the author established that the amount of drinking also acts as a factor in the increase of HTN risk.

The observed high prevalence of HTN amongst the urban population in this study is in line with Gupta (2015) [7]. This could be the impact of rapid urbanization and economic progress of rural populations with consequent changes in lifestyles. Most of the study participants are on a non-vegetarian diet, which could be an important factor for HTN considering the high fat and salt content [12]. HTN is also a major risk factor for the occurrence of ischemic heart diseases and stroke in later life, which stands to be the most important cause of morbidity and mortality according to the Global Burden of Disease studies [13]. The incidence of HTN in an individual depends on multiple interrelated and unrelated factors [14].

The predominant risk factors associated with HTN in the present study were high BMI (>25 kg/m2), smoking, alcoholism, and family history of HTN. This is per a meta-analysis conducted by Anchala et al. in 2014 [6]. The most common comorbidities in the study population were dyslipidemia, diabetes mellitus, cardiovascular diseases, and obesity, which is supported by the findings of the study by Alkaabi et al. (2019) [15]. As per the literature, insulin resistance and obesity play a central role in causing HTN and dyslipidemia [16]. Very few Indian studies have been published on the correlation between screen time exposure and HTN. In the present study, more than three hours of screen use of computers and smartphones was suggested by 32.9% and 67% of patients, respectively.

In the study patients, 68.79% were non-diabetic and the blood pressure (BP) goal was 140/90 mm Hg as per the older guidelines. The new guidelines suggest the BP goal of 130/80 mm Hg for all patients with HTN. According to the newer guidelines, 3437 (68.79%) of the study patients will be termed hypertensives even if they had achieved goal BP of 140/90 mm Hg [17-20]. Important factors considered for selecting a drug include the age of the patient, any concomitant comorbidity, safety, tolerability, efficacy, and cost. Combining two or more medications in modest doses may result in synergism and fewer negative effects. Since the therapy for HTN control is lifelong, fixed-dose combinations with longer half-lives could be considered to maintain patients’ compliance [21].

Amongst antihypertensive therapy, monotherapy was prescribed more than combination therapy, which aligned with the findings of Vashishtha et al. (2018) [22]. However, Datta (2016) [1] and Malhotra et al. (2001) [23] suggested that a combination of drugs was more frequently prescribed than a single drug. There is a growing recognition that angiotensin-converting enzyme inhibitors or angiotensin receptor blockers, calcium antagonists, or thiazide diuretics can be used as first-line therapy for HTN [24]. Evidence also supports the use of combination medication therapy rather than monotherapy for a greater synergistic effect on lowering blood pressure, offsetting side effects, and improving adherence to a drug regimen. Furthermore, appropriate antihypertensives have shown benefits in terms of reducing cardiovascular mortality and morbidity [25-27].

Limitations

This being a single-visit study, the follow-up for the disease progression and the compliance of the treatment were noted as per the knowledge of the reporting individual. The study reported the clinician’s perspective on the approach to HTN management wherein the patients’ perspective for the non-adherence to the drug regimen could be subjected to the implementation of suitable measures.

## Conclusions

In this cross-sectional study, conducted for the evaluation of trends in HTN management, we have concluded that monotherapy in the form of amlodipine/telmisartan is prescribed more commonly. Amongst antihypertensive combinations, a combination of amlodipine + chlorthalidone was most commonly prescribed, followed by amlodipine + metoprolol. Amongst other drugs used in patients with comorbidities, the most commonly prescribed drugs were metformin (antidiabetics), rosuvastatin (antilipidemic), and Ecosprin (antiplatelet). Despite the advances in HTN management and emphasis on patient education, there is a scope for creating implemented awareness among the patients. Management of HTN can be improved by imparting patient education and awareness about the need for medication compliance, lifestyle modifications, and regular follow-up clinic visits.
